# P-783. Can We Use the Cefazolin (CFZ) Urine-Specific Breakpoint to Predict Cephalexin Susceptibility for Klebsiella oxytoca (Kox) Urinary Tract Infection (UTI)?

**DOI:** 10.1093/ofid/ofaf695.994

**Published:** 2026-01-11

**Authors:** Sari B Cohen, Heather L Cox, Lindsay E Donohue, Amy Mathers, Stacy Park

**Affiliations:** Wellstar MCG Health Medical Center , Augusta, GA; University of Virginia Health, Charlottesville, Virginia; University of Virginia Health, Charlottesville, Virginia; University of Virginia, Charlottesville, VA; University of Virginia Medical Center, Charlottesville, VA

## Abstract

**Background:**

The Clinical and Laboratory Standards Institute (CLSI) recommends CFZ susceptibility (MIC ≤ 16 µg/mL) as a surrogate to predict select oral cephalosporin activity against *E. coli*, *Klebsiella pneumoniae*, and *Proteus mirabilis* for treatment of uncomplicated UTI (uUTI). Although there are limited clinical data, our institution applied this breakpoint to *Kox* until Aug 2023. This study assessed the real-world efficacy of cephalexin for *Kox* UTI utilizing the higher CFZ urine-specific breakpoint.

**Methods:**

This was a retrospective review of adults with ≥ 10^4^ CFU/mL *Kox* identified from urine cultures obtained in the University of Virginia Medical Center emergency department. Patients were included if prescribed cephalexin and excluded if given > 1 dose of an active parenteral antibiotic. The primary outcome was clinical failure within 30 days, defined as meeting ≥ 1 of 3 criteria: return to care or change in antibiotic due to persistent symptoms, or UTI recurrence with the same organism. Treatment success by MIC was also evaluated. CFZ susceptibility was determined via VITEK 2, where the lower limit of the calling range was ≤ 4 µg/mL, precluding assessment of interpretation using the CLSI CFZ breakpoint for *Kox* (MIC ≤ 2 µg/mL).

**Results:**

Between Jul 2017-Aug 2023, 189 *Kox* urine cultures were obtained from 163 unique patients, of which 28 met inclusion criteria. Patients were categorized as complicated UTI (39%, n=11), uUTI (32%, n=9), or acute pyelonephritis (29%, n=8). Clinical failure at 30 days occurred in 4 (14%) patients; two with uUTI. MICs among patients with clinical failure were: ≤ 4 (n=2), 8 (n=1), and ≥ 64 µg/mL (n=1). MICs for those with treatment success were: ≤ 4 (n=12), 8 (n=10), 16 (n=1), and ≥ 64 µg/mL (n=1).

**Conclusion:**

In this review, 23 of 26 (88%) patients were successfully treated with cephalexin for UTI caused by *Kox* with a CFZ MIC ≤ 16 µg/mL. At least 48% of these MICs should have been interpreted as not susceptible by CLSI criteria. There was a low rate of clinical failure, despite including patients with complicated UTI and acute pyelonephritis. Although larger comparative studies would be warranted, exploring the application of the CFZ urine-specific breakpoint for *Kox* should be considered.

**Disclosures:**

Lindsay E. Donohue, PharmD, BCIDP, Fingerpaint Inc.: Advisor/Consultant

